# The ESCRT-Related ATPase Vps4 Is Modulated by Interferon during Herpes Simplex Virus 1 Infection

**DOI:** 10.1128/mBio.02567-18

**Published:** 2019-03-05

**Authors:** Jorge Ruben Cabrera, Richard Manivanh, Brian J. North, David A. Leib

**Affiliations:** a Department of Microbiology and Immunology, Geisel School of Medicine, Dartmouth College, Lebanon, New Hampshire, USA; University of Pittsburgh School of Medicine; University of California, Irvine

**Keywords:** ESCRT, autophagy, herpes simplex virus, interferons, neuroimmunology

## Abstract

Interferons (IFNs) and autophagy are critical neuronal defenses against viral infection. IFNs alter neuronal autophagy by promoting the accumulation of IFN-dependent LC3-decorated autophagic structures, termed LC3 clusters. Here, we analyzed LC3 clusters in sensory ganglia following herpes simplex virus 1 (HSV-1) infection. In the vicinity of acutely infected neurons, antigen-negative neurons contained structures resembling accumulated autophagosomes and autolysosomes that culminated in LC3 clusters. This accumulation reflects a delayed completion of autophagy. The endosomal sorting complexes required for transport (ESCRT) machinery participates in autophagosome closure and is also required for HSV-1 replication. In this study, our results showed that HSV-1 infection *in vivo* and in primary neurons caused a decrease in Vps4 (a key ESCRT pathway ATPase) RNA and protein with concomitant Stat1 activation and LC3 cluster induction. We also observed that IFNs were sufficient to decrease RNA and protein levels of Vps4 in primary neurons and in other cell types. The accumulation of ubiquitin was also observed at the LC3 cluster sites. Together, our results show that IFNs modulate the ESCRT machinery in neurons in response to HSV-1 infections.

## Abstract (precis)

Neurons rely on IFNs and autophagy as major defenses against viral infections, and HSV must overcome such defenses in order to replicate. In addition to controlling host immunity, HSV must also control host membranes in order to complete its life cycle. HSV uses the host ESCRT membrane scission machinery for viral production and transport. Here we present evidence of a new IFN-dependent mechanism used by the host to prevent ESCRT subversion by HSV. This activity also impacts the dynamics of autophagy, possibly explaining the presence of recently described LC3 clusters in the HSV-infected nervous system. The induced accumulations of ubiquitin observed in these LC3 clusters resembled those observed in certain neurodegenerative diseases, suggesting possible mechanistic parallels between these conditions.

## INTRODUCTION

Herpes simplex viruses 1 and 2 (HSV-1 and HSV-2) replicate at skin and mucosal sites and invade sensory neurons (1). HSV particles are then retrogradely transported to the cell bodies of these neurons within sensory ganglia, wherein latency is established (1, 2). Latency allows the virus to evade immunity, persist lifelong, and periodically reactivate to spread in the population (1, 2). Latency and immune evasion are keys to the success of the herpesvirus family (3). HSV causes diseases that range from cold sores to more severe outcomes such as keratitis, encephalitis, and chronic pain (4). In addition, the presence of HSV-1 in the central nervous system (CNS) has been associated with neurodegenerative diseases (5).

Interferons (IFNs) and autophagy are key neuronal defenses against HSV-1 (6–8). Upon HSV-1 infection, neurons secrete and respond to type I IFNs (9), which activate the JAK-STAT pathway (10) and promote antiviral IFN-stimulated gene (ISG) expression (11). Macroautophagy (here autophagy) is important for the elimination of microorganisms (12). In brief, autophagy begins when cytosolic material is engulfed into double-walled autophagosomes, which fuse with lysosomes to form autolysosomes. Contents are then degraded by lysosomal enzymes (13). Neuronal autophagy is critical for development, differentiation, clearance of misfolded proteins, and pathogen defense (14). Importantly, type I IFN and autophagy are connected: type I IFN increases autophagy rates (15) and autophagy is required for type I IFN activation (16). IFN-γ is the only member of the type II IFNs. IFN-γ activates the JAK-STAT pathway in a different manner than type I IFNs, resulting in the transcription of type I IFN-overlapping and nonoverlapping ISGs (11). IFN-γ increases levels of autophagy during the antiviral response (17, 18), and autophagy is essential for antigen presentation and the development of the adaptive immune response (16).

A key pathway for both virus infectivity and host response is the endosomal sorting complexes required for transport (ESCRT) machinery. ESCRT promotes budding and partitioning of membranes during vesicle formation through reverse topology membrane scission, in which the cytosolic contents remain within the lumen of the vesicle (19). There are 5 ESCRT subgroups. ESCRT-0, ESCRT-I, and ESCRT-II complexes coordinate ESCRT assembly, membrane deformation, and cargo sorting. ESCRT-III and the vacuolar protein sorting 4 (VPS4) complexes catalyze membrane fission and ESCRT disassembly (20). ESCRT-III and VPS4 mediate the closure of autophagosomes, *in vitro* and *in vivo* (21–23). Many enveloped viruses require ESCRT machinery components for viral budding. HIV-1 subverts ESCRT-III/VPS4 machinery (23), and HSV-1 uses VPS4 and ESCRT-III for viral production, transport, envelopment, and nuclear egress (24–28).

HSV infection and IFN activation induce LC3-decorated autophagic structures in sensory neurons known as LC3 clusters (29). In this study, we characterized kinetics of LC3 clusters, IFN activation, and completion of autophagy in HSV-1-infected trigeminal ganglia (TG). We determined that LC3 clusters are structures resembling accumulations of autophagosomes and oversized autolysosomes *in vivo*. Given the similarities of LC3 clusters to accumulated autophagosomes in ESCRT-III/VPS4-deficient models (22, 30), we tested the hypothesis that IFNs control HSV-1 infection through Vps4 and other ESCRT pathway members. Vps4 RNA and protein levels were decreased in TG of HSV-infected mice, congruent with the presence of LC3 clusters and Stat1 activation. Using primary adult mouse TG neurons, we showed reduced Vps4 in HSV-1 antigen-negative neurons within infected cultures. We also showed that IFNs are sufficient to induce a decrease in RNA and protein levels of Vps4 and to alter ESCRT-III proteins. Decreased protein levels of Vps4 upon IFN stimulation were also observed in fibroblasts. Finally, we observed that LC3 clusters in primary cultures were proximal to areas of high levels of ubiquitin. Taken together, these results show that IFN-induced alterations of the ESCRT machinery in neurons likely act as a defense against HSV-1 infection.

## RESULTS

### LC3 clusters *in vivo* likely result from stalled IFN-induced autophagy.

**FIG 1:**
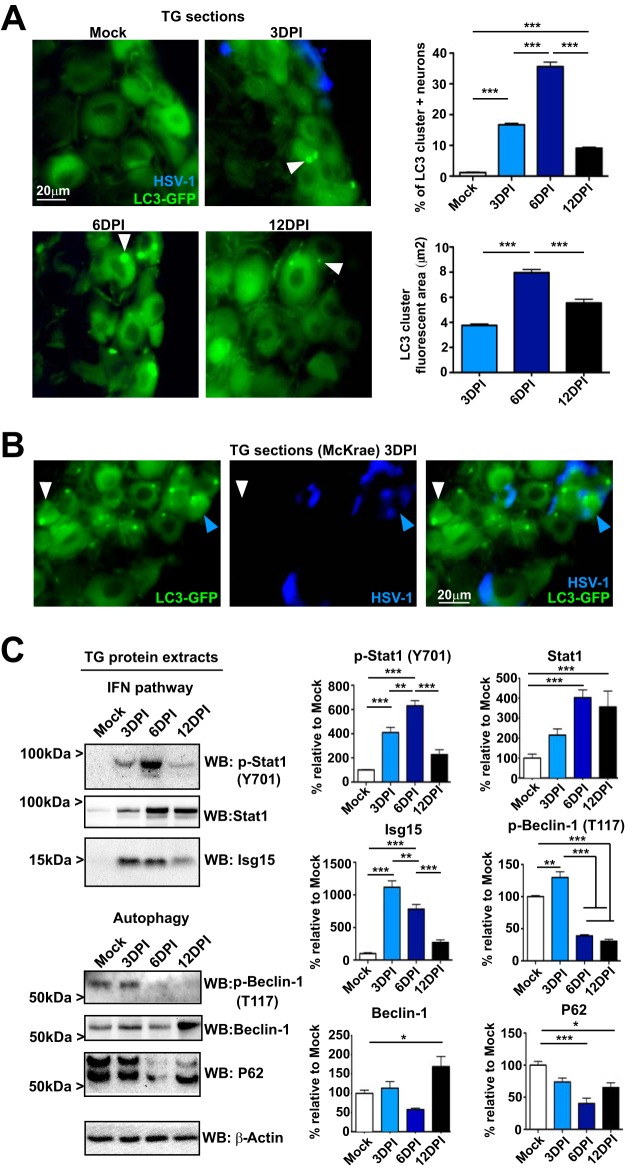
Time course of IFN signaling, autophagy, and presence of LC3 clusters in the TG after HSV-1 corneal infection. (A) (Left) Representative images of immunofluorescent microscopy using TG cryosections from corneally infected LC3-GFP^+/−^ mice (1 × 10e6 PFU/eye, HSV-1 strain 17) during the indicated times. LC3-GFP is in green, and detection of polyclonal antibody raised against HSV-1 is in blue. White arrowheads indicate representative LC3 clusters. (Upper right) Quantification of presence of LC3-GFP clusters in the ophthalmic branch of the TG. *n* = 6 TG from 6 mice, performed in two independent experiments. ***, *P* < 0.001. (Lower right) Quantification of size of LC3-GFP clusters in the ophthalmic branch of the TG. *n* > 100, performed in two independent experiments. ***, *P* < 0.001. (B) Representative image of immunofluorescent microscopy using TG cryosections from corneally infected LC3-GFP^+/−^ mice (1 × 10e6 PFU/eye, HSV-1 McKrae) 3 dpi. LC3-GFP is shown in green, and HSV-1 is in blue. White arrowheads indicate a representative LC3 cluster in an antigen-negative neuron. Blue arrowheads indicate an LC3 cluster in an antigen-positive neuron. (C) (Left) p-Stat1 (Y701), Stat1, Isg15, p-Beclin-1 (T117), Beclin-1, and P62 were analyzed by WB using TG protein extracts from infected LC3-GFP^+/−^ mice (1 × 10e6 PFU/eye, HSV-1 strain 17) during the time indicated. (Right) Quantification of WBs normalized to β-actin. Each protein analyzed was normalized to its own β-actin WB. *n* = 6 TG from 6 different mice, performed in two independent experiments. *, *P* < 0.05; **, *P* < 0.01; ***, *P* < 0.001.

LC3 clusters accumulate mainly in neurons in proximity to HSV-infected neurons (29). To establish the kinetics of these clusters, we used LC3-GFP^+/−^ mice in which LC3 is fused to GFP (31, 32). Transgenic cells display a cytoplasmic GFP haze, but autophagosome-bound GFP-LC3 manifests as distinct GFP puncta (0.5 to 1 μm), indicative of autophagy (29). We have defined LC3 clusters as accumulations of LC3-GFP of >2 μm^2^. LC3-GFP^+/−^ mice were infected with HSV-1 and analyzed for LC3 clusters and HSV-1 antigen expression (Fig. 1A). Mock-infected sections showed only sporadic LC3 clusters. At 3 days postinfection (dpi), HSV-1 antigen was detected in ophthalmic TG neurons and 15% of neurons were LC3 cluster positive (Fig. 1A). At 6 dpi, HSV-1 antigen detection was minimal but LC3 clusters increased up to 35% of ophthalmic branch neurons. At 12 dpi, HSV-1 antigens were absent and LC3 clusters were detected in <10% of neurons but remained significantly above mock-infected levels. LC3 cluster total fluorescent area mimicked this temporal pattern, averaging 4 µm^2^ at 3 dpi and 8 µm^2^ at 6 dpi and diminishing to 6 µm^2^ at 12 dpi (Fig. 1A).

LC3 cluster-positive neurons observed in Fig. 1A were HSV-1 antigen negative, in agreement with our previous report (29). However, some of the antigen-negative neurons could be HSV-1-infected neurons that are below the threshold of detection by immunofluorescence. To test whether LC3 clusters may occur in infected neurons, we performed corneal infection using HSV-1 strain McKrae. McKrae is more neuroinvasive than strain 17, facilitating HSV-1 detection. As seen with strain 17, LC3 cluster-positive neurons were almost entirely antigen negative 3 dpi with McKrae (Fig. 1B, white arrowheads). However, we were able to find occasional LC3 clusters in HSV-1-positive neurons (Fig. 1B, blue arrowheads). This result confirms that LC3 clusters are formed mainly in antigen-negative neurons but that HSV-1 antigens and LC3 clusters occasionally coexist in neurons.

Protein extracts from infected LC3-GFP^+/−^ mouse TGs were analyzed by Western blotting (WB) to track IFN pathway and autophagy activation and completion. Bulk TG extracts from LC3-GFP^+/−^ mice represent mainly noninfected tissue, since <2% of TG neurons innervate the cornea (33) and the C57/BL6 mouse background is relatively resistant to HSV infection (34, 35). Stat1 is activated by phosphorylation at tyrosine 701 (referred to here as p-Stat1) following IFN stimulation. TG p-Stat1 levels were significantly elevated 3 dpi, in agreement with type I IFN expression kinetics (36) (Fig. 1C). p-Stat1 peaked at 6 dpi, concomitant with the expected migration and activation of CD4^+^ and CD8^+^ cells and presence of IFN-γ in the infected TG (37). By 12 dpi, p-Stat1 had returned to background levels (Fig. 1C). We also measured protein levels of Stat1, which, as expected (11), were increased at 6 dpi and remained elevated at 12 dpi (Fig. 1C). Interferon-stimulated gene 15 (Isg15), a downstream effector of Stat1 signaling, showed a similar pattern as p-Stat1 but with a slightly earlier peak (Fig. 1C). To track autophagy, we measured activation/phosphorylation of the key autophagy protein Beclin-1 (38) on threonine 117 (p-Beclin-1) which activates it in a death-associated protein kinase (DAPK)- and IFN-dependent manner (17, 18). As expected, mock extracts showed significant p-Beclin-1, consistent with a high basal autophagy in neurons (39). At 3 dpi, p-Beclin-1 levels increased but unexpectedly dropped to levels below that of mock-infected cells at 6 and 12 dpi (Fig. 1C). We also measured protein levels of Beclin-1 which were steady state out until 12 dpi, at which point they were significantly increased (Fig. 1C). Finally, we analyzed autophagosome maturation by measuring levels of the autophagy adaptor protein P62, which decreases as autophagy is completed (40). P62 levels were comparable to mock-infected TG 3 dpi but were then significantly decreased at 6 and 12 dpi (Fig. 1C). At 12 dpi, a change in the band patterning of P62 was also observed; the lower band appeared stronger than the upper band, consistent with changes in P62 phosphorylation (41). Together, these results showed that LC3 clusters are transient structures that are proximal to, but not coincident with, infected HSV-1 neurons. We also observed a biphasic autophagic response with increases in p-Beclin-1 at 3 dpi while maturation of autophagy (P62 reduction) was delayed until6 to 12 dpi. The pattern of p-Beclin-1, Beclin-1, and P62 kinetics supports the hypothesis that LC3 clusters result from IFN-induced stalled autophagy.

### LC3 clusters *in vivo* are consistent with accumulated autophagosomes and autolysosomes.

**FIG 2:**
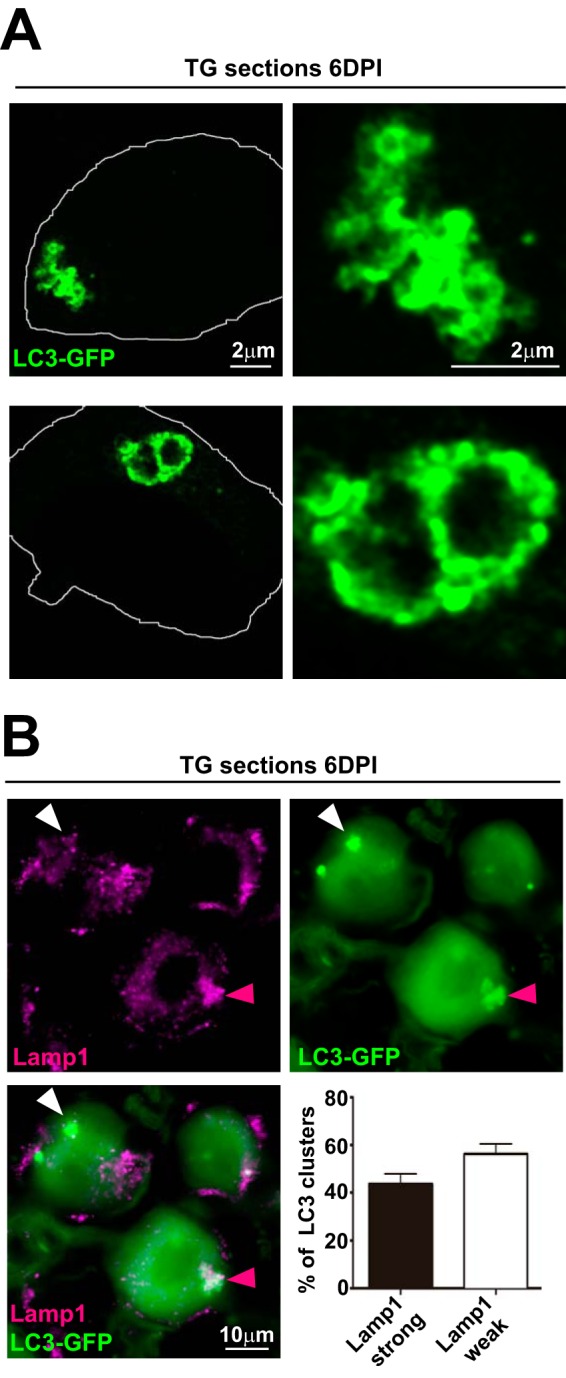
LC3 clusters in the TG after HSV-1 corneal infection are aggregations of autophagosomes and oversized autolysosomes. (A) Representative images of cluster morphologies taken with an Airyscan (Zeiss) confocal microscope showing immunofluorescent images of TG cryosections from infected LC3-GFP^+/−^ mice (1 × 10e6 PFU/eye, HSV-1 strain 17) at 6 dpi. LC3-GFP is in green. (B) Representative immunofluorescence images of TG cryosections from infected LC3-GFP^+/−^ mice (1 × 10e6 PFU/eye, HSV-1 strain 17) at 6 dpi. LC3-GFP is in green, and Lamp1 is shown in magenta. White arrowhead indicates representative Lamp1-weak LC3 cluster. Magenta arrowhead indicates representative Lamp1-strong LC3 cluster. Quantification of costained LC3-GFP clusters and Lamp1 in the ophthalmic branch; *n* = 11, performed in two independent experiments.

To further examine LC3 clusters *in vivo*, we performed high-resolution (AiryScan; Zeiss) confocal microscopy on TG sections from infected LC3-GFP^+/−^ mice at 6 dpi (Fig. 2A). LC3 clusters had two distinct morphologies. Some clusters appeared as accumulated GFP spheroids (closed or open) with sizes from 0.5 to 1 μm, compatible with autophagosomes and phagophores (Fig. 2A, upper panels). Other clusters appeared as irregular oversized spheres (>1 μm) (Fig. 2A, lower panels), consistent with aggregations of large autolysosomes resulting from lysosomal fusion with autophagosomes. To test these hypotheses, we probed TG sections for the lysosome marker Lamp1 by immunofluorescence (Fig. 2B). More than half of the LC3 clusters showed weak costaining with Lamp1, consistent with the hypothesis that these clusters are accumulated, early autolysosomes (Fig. 2B, white arrowhead). As expected, the remaining LC3 clusters showed strong costaining with Lamp1, suggesting that these clusters are more mature autolysosomes (Fig. 2B, magenta arrowhead). These results demonstrate that LC3 clusters *in vivo* show structures similar to accumulated autophagosomes and oversized autolysosomes.

### Vps4 is decreased in HSV-1-infected TG.

**FIG 3:**
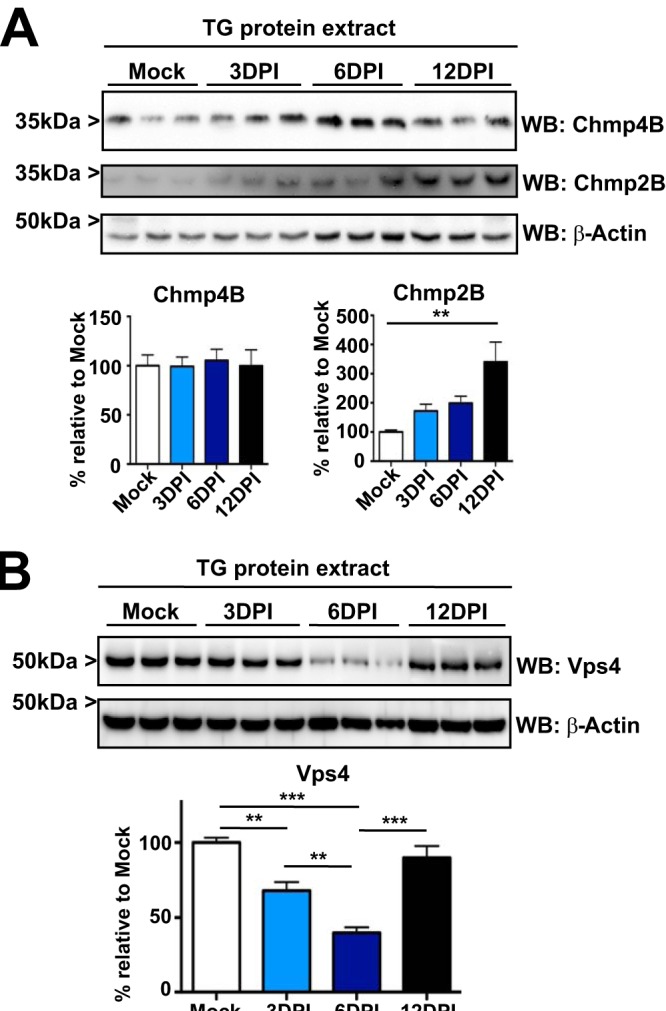
The ATPase Vps4 is reduced in the TG after HSV-1 corneal infection. (A) ESCRT-III proteins Chmp4B and Chmp2B were analyzed by WB using TG protein extracts from corneally infected LC3-GFP^+/−^ (1 × 10e6 PFU/eye, HSV-1 strain 17) mice and analyzed at the indicated times. Each line corresponds to a single TG. Graph shows quantification of WBs normalized to β-actin; *n* = 6 TG from 6 mice, performed in two independent experiments. **, *P* < 0.01. (B) Vps4 was analyzed by WB using TG protein extracts from infected LC3-GFP^+/−^ mice (1 × 10e6 PFU/eye, HSV-1 strain 17) at the indicated times. Each line corresponds to a single TG. Graph shows quantification of WBs normalized to β-actin; *n* = 9 TG from 9 different mice, performed in three independent experiments. **, *P* < 0.01; ***, *P* < 0.001. Vps4 antibody recognizes both Vps4A and Vps4B.

The precise mechanism of autophagosome closure remains obscure, but it seems likely that ESCRT-III and VPS4 complexes participate (42). In a variety of systems, modulation of ESCRT-III members (especially CHMP2B and 4B) and VPS4 results in autophagosome accumulation in neurons and other cells (21, 22, 30). Intriguingly, reduction of ESCRT-III and VPS4 activity also limits HSV-1 replication (24–28). This led us to hypothesize that IFNs may decrease ESCRT-III and/or Vps4 and stimulate neuronal antiviral defense. This decrease of ESCRT-III and/or Vps4 would also limit completion of autophagy, resulting in accumulation of autophagic structures and LC3 clusters. To test this hypothesis, we performed WB on protein extracts from TG of HSV-1-infected mice probing for Chmp2B and Chmp4B (Fig. 3A) and Vps4 (Fig. 3B). The core protein Chmp4B did not change significantly, but Chmp2B levels increased out to 12 dpi (Fig. 3A). In mammalian cells, the Vps4 complex is formed by oligomerization of two independent paralogues, Vps4A and Vps4B (43, 44). Vps4A and Vps4B share ∼80% amino acid sequence homology. We used an antibody capable of recognizing both isoforms. Surprisingly, Vps4 was significantly decreased at 3 dpi and 6 dpi but was comparable to mock levels at 12 dpi (Fig. 3B). These results show that Vps4, a key component of the autophagosome closure machinery, was decreased with comparable kinetics to phosphorylation of Stat1 (Fig. 1B) and presence of LC3 clusters (Fig. 1A).

### HSV-1 antigen-negative neurons show low levels of Vps4 in infected TG primary culture.

**FIG 4:**
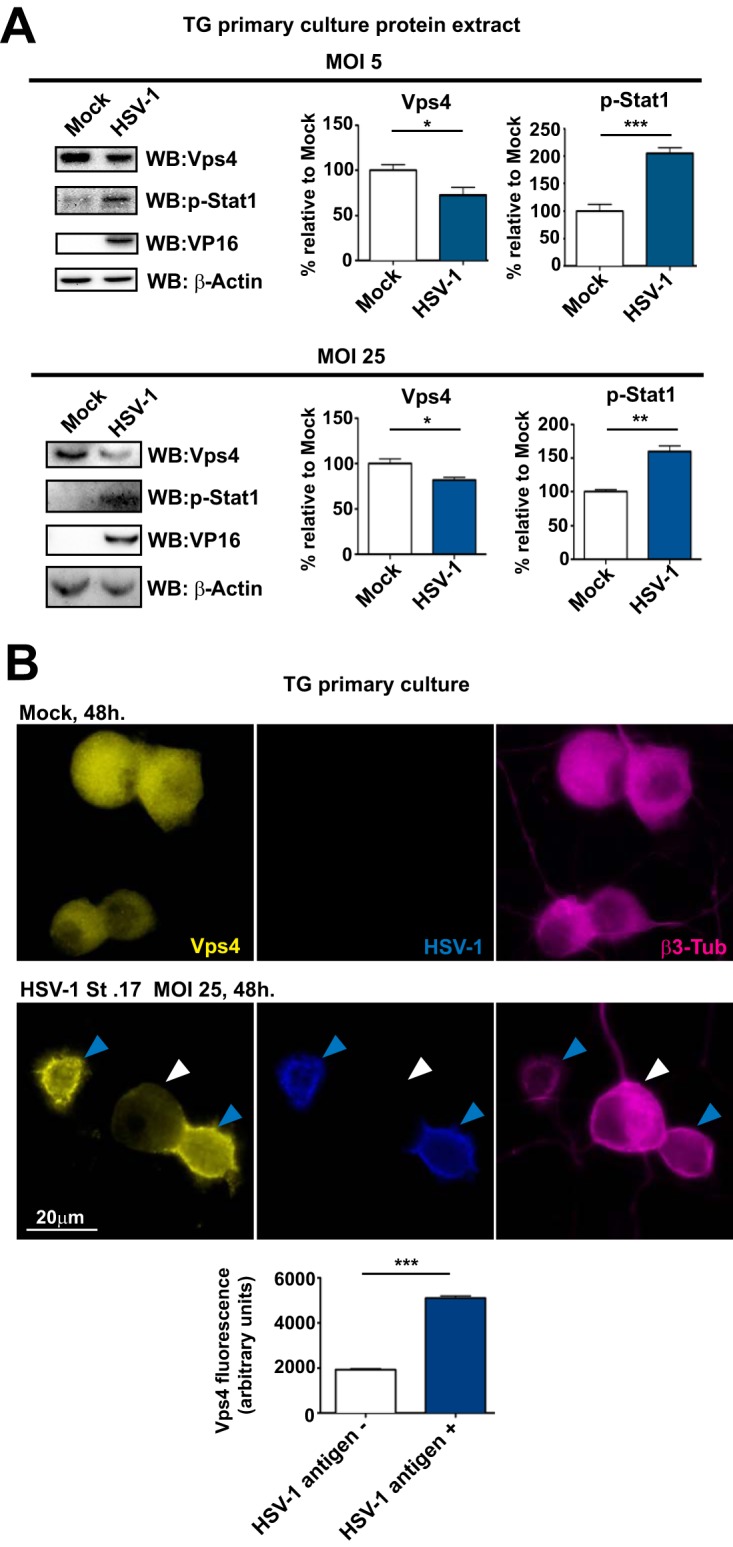
Vps4 is decreased in HSV-1 antigen-negative TG neurons. (A) Top, MOI of 5. (Left panel) WB of Vps4, p-Stat1, and VP16 from primary TG cultures of LC3-GFP^+/−^ mouse neurons which were mock infected or infected with HSV-1 strain 17 for 48 h. (Right panels) Quantification of WBs normalized to β-actin; *n* = 6, performed in two independent experiments. *, *P* < 0.05; ***, *P* < 0.001. Bottom, MOI of 25. (Left panel) WB of Vps4, p-Stat1, and VP16 from primary TG cultures of LC3-GFP^+/−^ mouse neurons which were mock infected or infected with HSV-1 strain 17 for 48 h. (Right panels) Quantification of WBs normalized to β-actin; *n* = 6, performed in two independent experiments. *, *P* < 0.05; **, *P* < 0.01. (B) Representative images of immunofluorescent microscopy of TG LC3-GFP^+/−^ mouse neurons. Mock-infected neurons are shown in upper panels. Neurons infected with HSV-1 strain 17 at an MOI of 25 for 48 h are shown in lower panels. Vps4 is in yellow, a polyclonal antibody raised against HSV-1 is shown in blue, and β3-tubulin is in magenta. The white arrowhead points to an HSV-1 antigen-negative neuron. Blue arrowheads point to HSV-1 antigen-positive neurons. Graph shows Vps4 fluorescence intensity in infected cultures for HSV antigen-negative (-) or -positive (+) neurons (β3-tubulin stained); *n* > 500 neurons were counted for each condition. The experiment shown is representative of two independent experiments; ***, *P* < 0.001.

*In vivo*, we observed a marked decrease of Vps4 levels in HSV-1-infected TG, yet relatively few TG neurons are infected with HSV following corneal challenge (33). It seems most likely, therefore, that our observed decrease in Vps4 resulted from a paracrine IFN-dependent antiviral response. To address this, we cultured primary adult mouse TG neurons, infected them with HSV-1 at MOIs of 5 and 25, and performed WB analysis for Vps4 and Stat1 (Fig. 4A). Consistent with the *in vivo* data, at both MOIs, levels of Vps4 were decreased and levels of p-Stat1 were increased in HSV-1-infected neuronal cultures. Although Vps4 protein levels were decreased, we were concerned that this could be due to HSV-1 induction of host shutoff cellular protein synthesis (45). We therefore performed an immunofluorescence (IF) assay to analyze Vps4 modulation in infected and noninfected neurons (Fig. 4B). In uninfected neuronal cultures, Vps4 staining was strong, diffuse, and homogeneous, and all neurons showed similar levels of Vps4 (Fig. 4B, upper panels). We infected TG neurons at an MOI of 25. At this MOI, all neurons were likely infected, but infections progressed efficiently in some neurons (antigen positive) while infection was not detected in others (antigen negative). In infected cultures, Vps4 staining was significantly altered, showing an uneven pattern (Fig. 4B, lower panels). MFI analysis showed strong Vps4 fluorescence in neurons that were HSV antigen positive in these infected cultures (Fig. 4, graph). In contrast, in HSV-1 antigen-negative neurons, Vps4 staining was decreased compared to antigen-positive neurons (Fig. 4B, lower panels, white arrowhead). Since decreased Vps4 corresponded with LC3 clusters, this result is consistent with the presence of LC3 clusters in HSV-1 antigen-negative neurons (29). Interestingly, reduced Vps4 was observed in almost all HSV-1 antigen-negative neurons, regardless of LC3 clusters (data not shown). Together, these results support the notion that decreased Vps4 is a component of an antiviral response that is coincident with increased levels of p-Stat1.

### IFNs are sufficient to decrease Vps4 protein levels in primary TG cultures and in other cell types.

**FIG 5:**
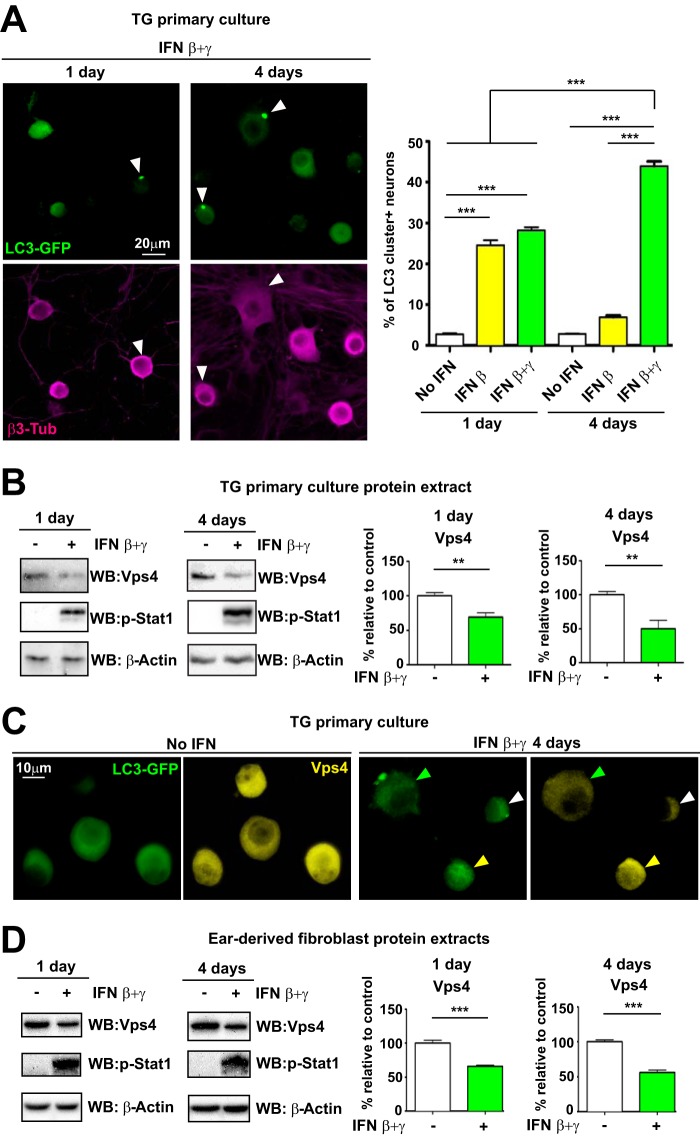
IFNs decrease Vps4 protein levels in TG mouse neurons and in other cell types. (A) (Left) Representative images from immunofluorescent microscopy of TG neurons from LC3-GFP^+/−^ mice in cultures not treated or treated with IFN β+γ at the indicated times. LC3-GFP is shown in green, and β3-tubulin is in magenta. White arrowheads point to representative LC3 clusters. (Right) Quantification of presence of LC3-GFP clusters in cultures treated with IFN-β or IFN β+γ; *n* = 8 (>1,000 neurons were counted for each condition). The experiment shown is representative of two independent experiments. ***, *P* < 0.001. (B) (Left panels) Vps4 and p-Stat1 were analyzed by WB using TG neurons of LC3-GFP^+/−^ mice treated with IFN β+γ at the times indicated. (Right panels) Quantification of WBs normalized to β-actin; *n* = 6, performed in two independent experiments. **, *P* < 0.01. (C) Representative images of immunofluorescent microscopy of TG LC3-GFP^+/−^ mouse neurons. Nontreated neurons are shown in left panels. Neurons treated for 4 days with IFN β+γ are shown in right panels. Vps4 is in yellow, and LC3-GFP is shown in green. The yellow arrowhead points to an LC3 cluster-negative Vps4-strong neuron. The white arrowhead points to an LC3 cluster-negative Vps4-weak neuron. The green arrowhead points to an LC3 cluster-positive Vps4-weak neuron. (D) (Left panels) Vps4 and p-Stat1 were analyzed by WB using ear-derived fibroblasts of LC3-GFP^+/−^ mice treated with IFN β+γ at the times indicated. (Right panels) Quantification of WBs normalized to β-actin; *n* = 6, performed in two independent experiments. ***, *P* < 0.01.

Our results showed an inverse relationship between levels of p-Stat1 and Vps4. In a previous report, we demonstrated that LC3 clusters were formed when neurons were treated with exogenous IFN-β (29). These LC3 clusters induced by IFN-β in culture peaked at 12 to 24 h posttreatment and decreased rapidly thereafter. *In vivo*, in contrast, LC3 clusters remained stable for several days, suggesting that there may be an additional factor which sustains or stabilizes LC3 clusters (Fig. 1). We hypothesized that IFN-γ may be this factor. In order to more closely mimic the *in vivo* situation, we stimulated neurons in primary culture with a combination of 100 IU/ml of IFN-β plus 50 IU/ml of IFN-γ (IFN β+γ) or with 100 IU/ml of IFN-β. In nontreated neurons, LC3 clusters were rarely found at any time tested (Fig. 5A, graph). After IFN-β treatment for 1 day, ∼25% of neurons were LC3 cluster positive (Fig. 5A, graph), and similar levels were found when neurons were stimulated with IFN β+γ (Fig. 5A, graph and left panels). As expected, levels of LC3 clusters dropped to basal levels following IFN-β treatment for 4 days (Fig. 5A, graph). In contrast, treatment with IFN β+γ resulted in a sustained increase in numbers of LC3 cluster-positive neurons relative to those treated with IFN β+γ for 1 day (Fig. 5A, graph and right panels). IFN β+γ treatment of TG neurons therefore recapitulates the sustained presence of LC3 clusters *in vivo*. We then analyzed these TG neurons by WB for Vps4 (Fig. 5B). Vps4 protein levels were decreased after 1 and 4 days of treatment with IFN β+γ compared to nontreated neurons (Fig. 5B). These results confirmed that IFNs reduced Vps4 protein levels. We probed for p-Stat1, which, as expected, was present in IFN β+γ-treated neurons at all time points tested (Fig. 5B), while protein levels of Chmp4b and Chmp2B were unchanged (data not shown).

We next examined TG primary neurons by IF for Vps4 upon IFN β+γ treatment for 4 days. While untreated neurons showed a strong, diffuse, and homogeneous Vps4 staining (Fig. 5C, left panels), treatment with IFN β+γ resulted in a general decrease of Vps4 staining (Fig. 5C, right panels). Three distinct patterns emerged: neurons negative for LC3 clusters with a strong Vps4 staining (Fig. 5C, right panels, yellow arrowhead), neurons negative for LC3 clusters with weak Vps4 staining (Fig. 5C, right panels, white arrowhead), and neurons positive for LC3 clusters with weak Vps4 staining (Fig. 5C, right panels, green arrowhead). It was noteworthy that all LC3 cluster-positive cells showed weak Vps4 staining. That said, not all Vps4-weak neurons were LC3 cluster positive. This suggests that TG neurons do not respond equally to IFN, which is expected since these neurons are heterogeneous (46). Finally, we wished to address whether the IFN-induced decrease of Vps4 is a neuron-specific response or represents a more broad-spectrum antiviral response. To test this, we treated a variety of primary cell types with IFN β+γ. Adult mouse TG-derived glia and fibroblasts did not show any change in Vps4 protein levels upon IFN β+γ treatment (data not shown). In contrast, IFN β+γ-treated adult mouse primary fibroblasts showed strong decreases in protein levels of Vps4 (Fig. 5D), although LC3 clusters were not detected in these cells (data not shown). These results show that treatment with IFN β+γ induced stable LC3 clusters and decreased Vps4 levels in TG neurons. Decreased Vps4 was also observed in fibroblasts in the absence of LC3 clusters.

### IFNs are sufficient to decrease Vps4A and Vps4B RNA levels in primary TG cultures.

**FIG 6:**
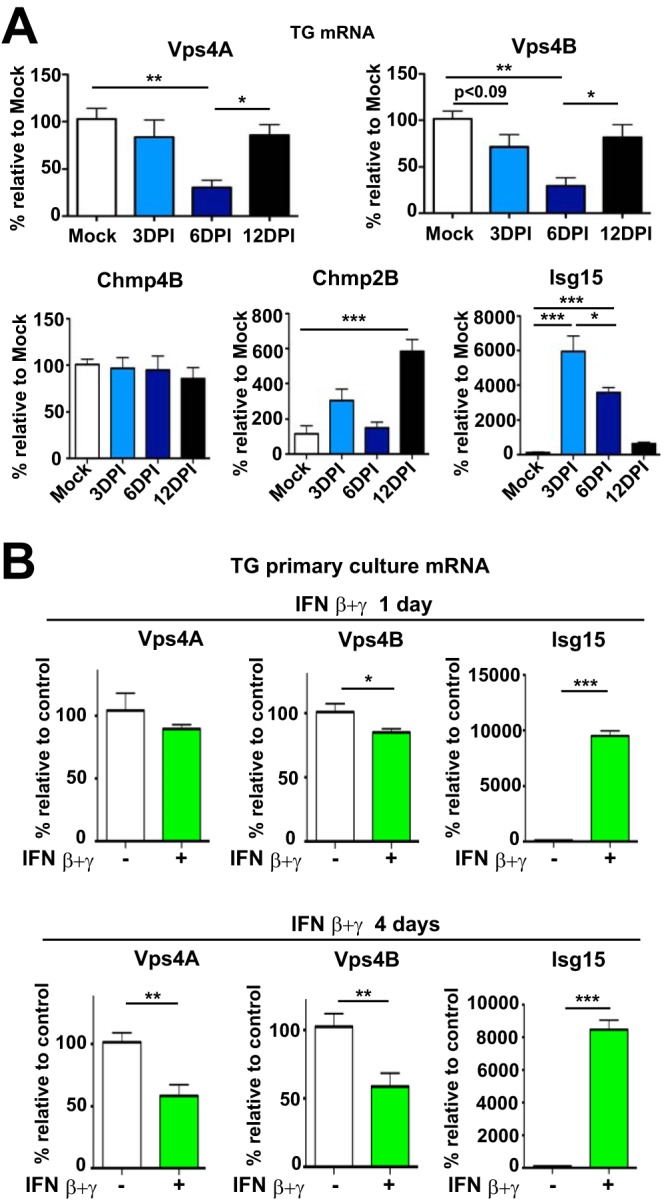
IFNs decrease Vps4A and Vps4B mRNA levels in TG mouse neurons. (A) mRNA levels of Vps4A, Vps4B, Chmp4B, Chmp2B, and Isg15 were analyzed by qPCR using TG mRNA from infected LC3-GFP^+/−^ mice during the indicated times. Quantification of Sybr Green signal was normalized to β-actin; *n* = 5 for all genes analyzed and all time points, except for Chmp2B Mock (*n* = 3). All samples were performed with two technical replicates. *, *P* < 0.05; **, *P* < 0.01; ***, *P* < 0.001. (B) mRNA levels of Vps4A, Vps4B, and Isg15 were analyzed by qPCR using TG primary neurons from LC3-GFP^+/−^ mice treated with IFN β+γ during indicated times. Quantification of Sybr Green signal was normalized to β-actin; *n* = 6 for all genes analyzed and all time points. All samples were performed with two technical replicates. *, *P* < 0.05; **, *P* < 0.01; ***, *P* < 0.001.

We further analyzed changes in Vps4 observed in TGs of HSV-1-infected animals by real-time qPCR analysis of Vps4 expression. *Vps4A* and *Vps4B* differ in their 5′ UTR at the mRNA level, allowing independent analysis of both genes. We isolated mRNA from TG of mice corneally infected with HSV-1 and found that Vps4A RNA levels were unchanged at 3 dpi, significantly decreased at 6 dpi, and comparable to mock levels at 12 dpi (Fig. 6A, upper left). In contrast, Vps4B RNA levels followed a similar pattern as Vps4A (Fig. 6A, upper right). We also analyzed RNA levels of ESCRT-III genes known to be involved in neuronal autophagy (Fig. 6A, lower left). Chmp4B RNA was unchanged during the course of the infection, while Chmp2B mRNA was significantly increased at 12 dpi (Fig. 6A, lower middle). This is in agreement with increased Chmp2B protein at the same time point (Fig. 3A). As an internal control, we monitored mRNA levels of Isg15 (Fig. 6A, lower right), consistent with the protein data (Fig. 1B). We confirmed these data by using mRNA from adult TG neurons treated with IFNs. There were no changes in RNA levels of Vps4A in IFN β+γ–treated TG neurons on day 1 (Fig. 6B, left upper), but Vps4B RNA was significantly decreased (Fig. 6B, upper middle). After 4 days of IFN β+γ treatment, RNA levels of both Vps4A and Vps4B were substantially decreased (Fig. 6B, lower left and lower middle, respectively). As a positive control, we observed expected significant increases in Isg15 RNA 1 and 4 days post-IFN β+γ treatment (Fig. 6B, upper right and lower right, respectively). These results show that IFNs can cause significant decreases in Vps4A and Vps4B RNA levels.

### IFNs alter Chmp4B in TG primary culture.

**FIG 7:**
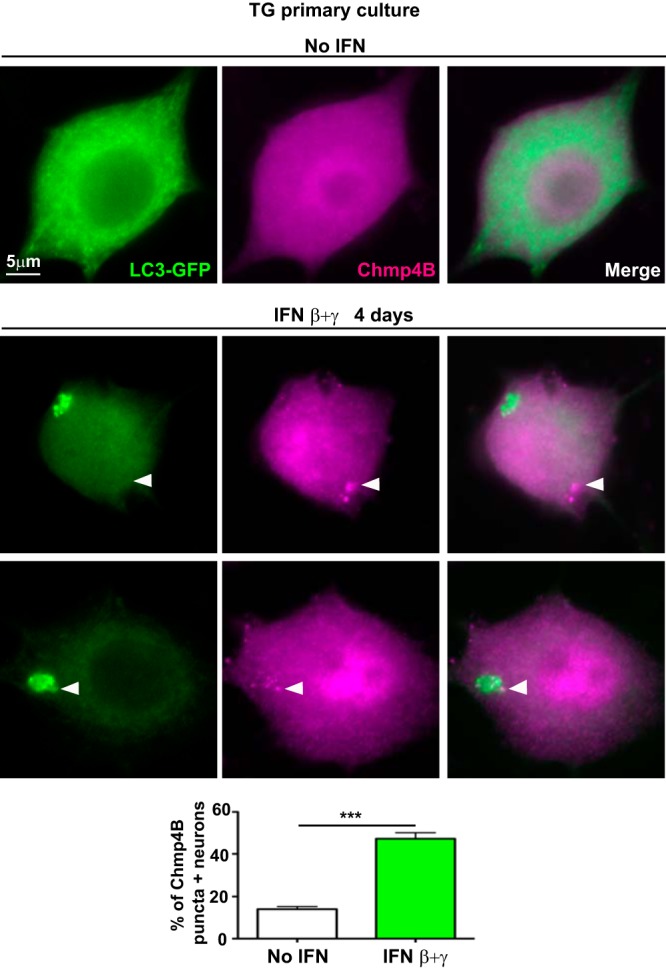
IFNs induce Chmp4B puncta in TG neurons. Representative images from immunofluorescence microscopy of TG neurons from LC3-GFP^+/−^ mice in culture not treated or treated with IFN β+γ for 4 days. LC3-GFP is shown in green and Chmp4B in magenta. White arrowheads point to Chmp4B puncta. Graph shows quantification of neurons containing Chmp4B-positive puncta in the cultures; *n* = 8 (>400 neurons were counted for each condition). The experiment shown is representative of two independent experiments. ***, *P* < 0.001.

ESCRT-III proteins, such as Chmp4B, remain soluble and monomeric prior to activation. When recruited, these proteins polymerize and form filaments that are key to membrane scission. The ATPase complex Vps4 provides energy during the last steps of membrane scission and allows depolymerization and recycling of ESCRT-III proteins (20). In addition, overexpression of ISG15 alters several ESCRT-III proteins, like CHMP4B, and ISGylation blocks their interaction with VPS4 (47, 48). Together, these data make it plausible that IFNs affect ESCRT-III protein activity in TG neurons. To test this, we treated mouse adult TG neurons with IFN β+γ and stained them for Chmp4B (Fig. 7). In nontreated TG neurons, Chmp4B was largely homogeneous (Fig. 7, upper panels), as expected for a soluble cytoplasmic protein (49). In IFN β+γ-treated neurons, however, we observed that ∼50% of the neurons exhibited accumulations of Chmp4B or “Chmp4B puncta” (Fig. 7, graph). Chmp4B puncta were not associated with LC3 clusters in some neurons (Fig. 7, middle panels), were observed in LC3 cluster-negative neurons (data not shown), and were in close proximity to LC3 clusters in other neurons (Fig. 7, lower panels). This is consistent with the hypothesis that Chmp4B puncta represent accumulated Chmp4B filaments within membranes of autophagic structures and other membrane-bound vesicles, as a result of limiting levels of Vps4.

### LC3 clusters contain ubiquitin in IFN-treated TG neurons.

**FIG 8:**
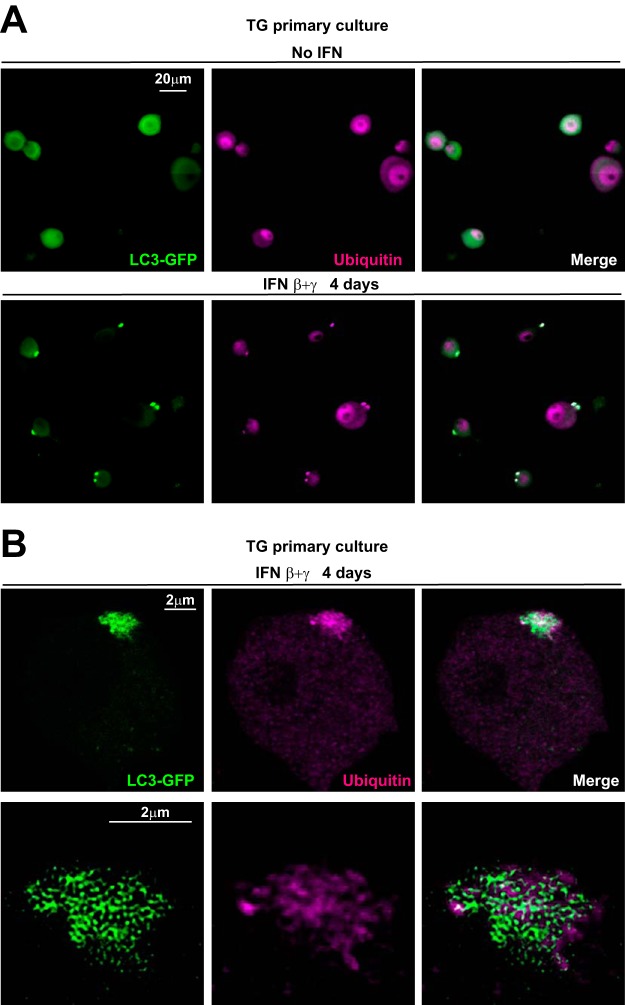
LC3 clusters are associated with ubiquitin accumulations. (A) Representative images from immunofluorescence microscopy of cultured TG neurons from LC3-GFP^+/−^ mice that were either untreated (upper row) or treated (lower row) with IFN β+γ for 4 days. LC3-GFP is in green; ubiquitin is shown in magenta. The experiment shown is representative of three independent experiments; >1,000 neurons were analyzed. (B) Representative stack from high-resolution (Airyscan, Zeiss) confocal microscopy of TG neurons from LC3-GFP^+/−^ mice in culture treated with IFN β+γ for 4 days (upper row). Lower row shows the magnification and deconvolution of the above LC3 cluster and ubiquitin accumulation. LC3-GFP is in green; ubiquitin is shown in magenta.

Transgenic *Drosophila* flies which overexpress dominant negative VPS4 show ubiquitin colocalized with LC3 cluster-like aggregates in the brain (22). Ubiquitinylation is a process involved in autophagy by which proteins and organelles are selectively tagged for degradation (13). If IFN delays the completion of autophagy, then LC3 clusters should colocalize with an accumulation of ubiquitin. To test this hypothesis, we examined the contents of LC3 clusters by IF microscopy using the P4D1 monoclonal antibody, which recognizes ubiquitin and polyubiquitinated and ubiquitinated proteins. By regular microscopy, we observed that following IFN β+γ treatment, all LC3 clusters were colocalized with ubiquitin (Fig. 8A). High-resolution (AiryScan; Zeiss) confocal microscopy showed that ubiquitinated proteins accumulated either inside or surrounding the LC3 clusters (Fig. 8B). These data suggest that LC3 clusters contain cellular material which is either undigested or about to be digested. These results demonstrated that LC3 clusters occur in close proximity to ubiquitin accumulation, comparable to the pattern observed in VPS4 dominant negative transgenic *Drosophila* (22). These data have important implications, as discussed below, for the functional significance of LC3 clusters.

## DISCUSSION

Herpesviruses and their host cells compete for the control of internal membranes in infected cells. ESCRTs are a group of proteins which regulate reverse topology membrane scission and are involved in many functions, including the formation of multivesicular endosomes and autophagosomes, among other functions (50). Due to their ability to reorganize internal membranes, many viruses commandeer ESCRT components during viral replication (23). In particular, HSV-1 uses VPS4 and several ESCRT-III proteins to rearrange cellular membranes for replication (24–28). In this study, we have presented evidence that IFNs modulate the ESCRT machinery in response to HSV-1 infection. Our results show that, upon HSV-1 corneal infection, levels of Vps4 are significantly decreased in the TG, in parallel with p-Stat1 activation and expression of Isg15. Additionally, in primary culture, IFN treatment reduces levels of Vps4 in adult TG sensory neurons. Therefore, reduction of Vps4 by IFN alters ESCRT-III protein function. Overexpressing dominant negative VPS4 is efficient in limiting HSV-1 production (24, 26, 27), consistent with the idea that host defenses utilize a similar antiviral strategy.

One of the functions of IFNs is to maintain control of host membranes upon infection (11). To do so, IFNs stimulate production of several membrane-modulating ISGs, such as tetherin, viperin, and IFITM (11). In addition, IFNs regulate ESCRT-III function through ISG15 (47, 48), a small molecule which acts in an ubiquitin-like manner (51) with strong antiviral properties against HSV and other viruses (52). Several CHMPs, including CHMP4B, can be covalently bound to ISG15 (47, 48). Upon conjugation, the ability of CHMPs to interact with VPS4 is reduced, thereby slowing the system and reducing its utility for viruses (47, 48). In these previous studies, however, ISG15 was associated with CHMPs and not with VPS4 (47, 48). Our finding that IFNs directly regulate Vps4 in neurons and in other cell types therefore adds a new and complementary layer of control of ESCRT machinery by IFNs. ISG15 conjugation was recently shown to be a broad nonspecific mechanism to destabilize newly synthesized viral proteins (53, 54). It is possible therefore that IFNs control the ESCRT machinery in two different ways, first in a nonspecific manner through ISG15 and second in a more ESCRT-specific manner by reducing levels of Vps4. In support of this hypothesis, we have recently observed that LC3 clusters are still present in the TG of HSV-1-infected Isg15-deficient mice (data not shown).

An important open question is how IFNs regulate Vps4. IFNs lead to Stat1 phosphorylation, translocation into the nucleus, and binding to DNA, promoting the transcription of ISGs (10, 11). However, some genes are repressed as a consequence of transcriptional changes mediated by Stat1 (55, 56), and this may be the case for Vps4A and Vps4B. Our results show that IFN treatment leads to decreased Vps4 RNA and protein. *Vps4A* and *Vps4B* are positionally conserved among mammals, consistent with the idea that their genomic position may be important for the control of their expression. Indeed, the *Vps4A* gene is close to a conserved Stat1 binding site (57, 58). We hypothesize that increased transcription of genes surrounding *Vps4A* upon IFN activation may interfere with *Vps4A* transcription. *Vps4B* is in the vicinity of the antiapoptotic gene *Bcl-2* (58), which is highly regulated by Stat1 and IFNs (59–61), and a similar mechanism may explain the IFN-induced decrease of *Vps4B* mRNA. Studies are ongoing to better understand the regulation of these important genes by IFN.

The initial goal of this study was to determine the molecular basis of neuronal LC3 cluster formation upon IFN stimulation (29). IFNs increase levels of autophagy (15, 16), and autophagy/xenophagy is a key antiviral defense in neurons against HSV-1 (7, 8, 29). As expected, some autophagy markers were increased rapidly upon HSV-1 infection of the TG with strong LC3 accumulation and increased levels of p-Beclin-1. However, the maturation of autophagosomes as measured by the degradation of P62 did not occur until 6 dpi. Vps4 and ESCRT-III regulate the closure of autophagosomes, and IFNs may therefore modulate autophagy in two different ways (21, 22, 30). First, it may increase levels of upstream autophagy. Second, it may reduce levels of Vps4 and reduce Chmp activity by ISGylation, thereby slowing the closure of autophagosomes. These data fit with the strong association of ubiquitin with LC3 clusters, which would mark undigested or soon-to-be digested cargo. We thus propose that LC3 clusters in neurons are the result of IFN-induced autophagy that is delayed in its maturation. This hypothesis presents LC3 clusters as a passive by-product of two activities, IFN-induced autophagy and IFN-induced decrease of Vps4. While we favor this hypothesis, it is also possible that LC3 clusters have a more active function. Since neurons have high levels of basal autophagy, increasing those levels further could jeopardize neuronal integrity and result in autophagic cell death (39). Slowing the completion of autophagy would be a safe manner to control the IFN-induced increase of autophagy. Another possibility is that LC3 clusters may be reservoirs for MHC-I loading. Neurons are polarized and immune-privileged cells, and unstimulated neurons do not express detectable levels of MHC-I. The IFN response to HSV-1 infection, however, induces both LC3 clusters (29) and increased levels of MHC-I in sensory neurons (62). It is plausible that LC3 clusters may accumulate proteins from distal sources in the neuron to digest them and facilitate the loading of peptides into MHC-I.

Finally, some authors have proposed that HSV-1 infections are associated with, and may even be causal to, neurodegenerative diseases (5, 63–65). Curiously, some mutations in autophagy-related genes and in the ESCRT-III protein CHMP2B lead to neurodegenerative diseases with accumulation of ubiquitin (66–69). Here, we present evidence showing that HSV-1-induced IFN decreases Vps4 and alters ESCRT-III activity, causing a delayed completion of autophagy and accumulation of ubiquitin. These changes appeared to be transient and associated with HSV-1 productive infection. HSV-induced LC3 clusters, however, resemble some neurodegenerative markers (14, 30). These similarities may fuel further speculation regarding the interplay of HSV-1 chronicity and degenerative diseases of the human central nervous system (5).

## MATERIALS AND METHODS

### Viruses and animals.

The wild-type HSV-1 strain 17 syn^+^ (GenBank accession no. NC_001806) and the wild-type HSV-1 strain McKrae (GenBank accession no. JX142173) (70) were propagated and plaqued on Vero cells using standard practices, as previously described (71).

Noboru Mizushima provided green fluorescent protein (GFP)-LC3 mice (31, 32). To maintain the colony of GFP-LC3 ^+/−^ mice, these were crossed with C57BL/6J mice purchased from Jackson Laboratories and bred in-house.

This study was carried out in strict accordance with the recommendations in the *Guide for the Care and Use of Laboratory Animals* of the National Research Council (72). The protocol was approved by the Dartmouth IACUC (permit number leib.da.1#2m13a). No surgery was performed, and all efforts were made to minimize suffering.

### Culture of adult mouse TG neurons.

TG neurons from adult mice were isolated and cultured as previously reported (73). Twelve-millimeter coverslips (for IF) or M24 wells (for WB) were coated with poly-d-lysine (BD Biosciences) at 100 μg/ml in Hanks balanced salt solution lacking calcium and magnesium (HBSS; HyClone) overnight. Surfaces were then washed three times with HBSS and coated with natural mouse laminin (Invitrogen) at a concentration of 18 μg/ml in HBSS overnight. TG neurons were isolated as described previously, with a few modifications (73). Mice 5 to 10 weeks old were euthanized using approved methods and transcardially perfused with phosphate-buffered saline (PBS; HyClone). TG were harvested and enzymatically digested in a solution consisting of 40 U/ml of papain (Worthington) in HBSS with 2.75 mM l-cysteine (Sigma) and 8% NaHCO_3_ diluted 1:1,000 (Sigma) for 20 min at 37°C on a rotator. This was followed by a similar incubation in a solution of 5 mg/ml of collagenase type II (Invitrogen) and 5.5 mg/ml of neutral protease (Worthington) dissolved in HBSS. TG were then triturated in Neurobasal-A (NB-A) working medium (Neurobasal-A (Invitrogen), 2% B27 (StemCell), and 1% penicillin-streptomycin (Pen-Strep; HyClone). The resulting homogenate was spun over a two-layer density gradient made with Percoll and NB-A working medium. Neurons were pelleted by a 10-min centrifugation at 1,300 × *g*, upper layers were discarded, and the pellet was washed three times in NB-A working medium. Neurons were resuspended in minimal volumes of NB-A complete medium, including the antimitotic 5-fluoro-2-deoxyuridine (FUDR; Sigma) for a minimum of 3 days prior to use. NB-A complete medium consisted of Neurobasal-A, 2% B27, 1% GlutaMAX (Invitrogen), 1% Pen-Strep, 50 ng/ml of nerve growth factor (NGF; Invitrogen), 50 ng/ml of glial cell-derived neurotrophic factor (GDNF; R&D Systems), and 50 ng/ml of Neurturin (R&D Systems). Three thousand six hundred neurons were seeded per 12-mm glass slide or M24 well and were grown for 2 or 3 days before treatments or infections.

### Ear-derived fibroblast isolation and culture.

Fibroblasts from adult mice were obtained through ear clippings and subsequently minced and digested in 1,000 U/ml collagenase type II (Invitrogen) followed by 0.05% trypsin (Cellgro). Resulting cell lysate was triturated and plated in 6-well plates in DMEM (HyClone) with 10% FBS, 1% nonessential amino acids, 1% GlutaMAX (Invitrogen), and 1% Pen-Strep.

### Treatments.

Mouse adult TG neurons and mouse adult ear-derived fibroblasts were treated with 100 U/ml of mouse IFN-β (PBL Interferon Source) and 50 U/ml of mouse IFN-γ (Miltenyi Biotec) or with 100 U/ml of mouse IFN-β (PBL Interferon Source) for the time indicated.

### HSV-1 infections. (i) *In vivo*.

Mice were anesthetized intraperitoneally with ketamine (90 mg/kg of body weight) and xylazine (10 mg/kg). Corneas were bilaterally scarified with a 25-gauge syringe needle, and virus was inoculated by adding 1 × 10^6^ PFU per eye in a 3-μl volume. To reduce pain caused at the cornea, mice were then injected with buprenorphine (0.41 mg/kg).

### (ii) *In vitro*.

Infections of TG primary cultures were performed at MOIs of 5 and 25 for 48 h. Cultures were incubated with the virus for 1 h to allow viral adsorption, and then medium was replaced.

### Western blots.

Extracts were prepared by homogenizing TG in ice-cold extraction buffer consisting of 20 mM HEPES, pH 7.4, 150 mM NaCl, 1% Triton X-100, cOmplete protease inhibitor cocktail tablets (Roche), and PhosStop phosphatase inhibitor cocktail (Roche). Samples were homogenized and centrifuged at 15,000 × *g* for 15 min at 4°C. The resulting supernatant was collected, and protein content was determined by Bradford assay. Ten to 30 micrograms of total protein was electrophoresed on an SDS-polyacrylamide gel (concentration ranged between 8% and 15% depending on size of the protein analyzed), transferred to a PVDF membrane, and blocked in PBS-T with 5% nonfat dry milk. Primary antibodies were incubated overnight. Membranes were developed using SuperSignal West Dura extended-duration substrate (Thermo Fisher Scientific) using an Alpha Innotech FluorChem Q imager. Every WB was stripped using Restore (Thermo Fisher Scientific) and rehybridized against β-actin for normalization.

### Antibodies. (i) IF.

Rabbit polyclonal anti-HSV-1 was purchased from Dako. Chicken anti-β3-tubulin was obtained from Millipore. Mouse monoclonal CHMP4B antibody (clone 13G12) was from Covalab. Antibodies against Lamp1 (clone 1D4B), VPS4 (clone E8), and ubiquitin and polyubiquitinated and ubiquitinated proteins (clone P4D1) were from Santa Cruz Biotechnology.

Secondary antibodies, all Alexa Fluor conjugated, were from Invitrogen: goat anti-rabbit 350 A-21068, goat anti-mouse 555 A-32727, goat anti-chicken IgY 647 A-21449, and goat anti-rat 647 A-21247.

### (ii) WB.

Rabbit polyclonal anti-p-Stat1 701 #9171 was purchased from Cell Signaling. Stat1 no. 610186 was from BD. Rabbit polyclonal anti-p-Beclin-1 threonine 119 in human sequence (threonine 117 in mouse sequence) was from Abgent. The antibody against P62 (NBP1-48320) was obtained from Novus Bio. Anti-β-actin (Poly6221) was from BioLegend. Mouse monoclonal anti-CHMP4B (clone 13G12) was from Covalab. The antibody against CHMP2B was purchased from R&D Systems. Monoclonal antibodies recognizing ISG15 (clone F9), BECN-1 (clone E8), VPS4 (clone E8), and anti-VP16 (1-21, sc-7545) were from Santa Cruz Biotechnology.

### Immunofluorescence microscopy. (i) Neuron cultures.

Cultures were fixed using 4% PFA in 0.1 M phosphate buffer (PB) for 10 min. Coverslips were washed three times with 0.1 M PB and incubated with 1% BSA and 1% Triton X-100 in 0.1 M PB for 1 h and then overnight with primary antibodies diluted in 1% BSA, 1% Triton X-100 in 0.1 M PB. The next day, coverslips were washed three times with 1% Triton X-100 in 0.1 M PB for 10 min each, and then coverslips were incubated with secondary antibodies diluted in 1% BSA, 1% Triton X-100 in 0.1 M PB for 2 h. Finally, coverslips were washed with 1% Triton X-100 in 0.1 M PB for 10 min and in 0.1 M PB for an additional 10 min. After the final wash, coverslips were mounted in FluorSave (Calbiochem). Coverslips were imaged using an automated AxioVision Observer Z1 (Zeiss) microscope. Random tile images were acquired for analysis, and images were analyzed using ZEN2012 or NIH Fiji software.

### (ii) Immunohistochemistry.

Mice were euthanized at the time point indicated by approved methods and transcardially perfused with PBS. TG were harvested and fixed *ex vivo* with 4% formaldehyde (PFA; Fisher Scientific) in 0.1 M PB for 3 h. They were washed 3 times in 0.1 M PB and incubated in 15% sucrose overnight at 4°C and in 30% sucrose overnight at 4°C. Cryoprotected ganglia were embedded in Tissue-Tek OCT compound (Sakura). The tissue was sectioned using a Leica CM1860 cryostat into 15-μm transverse plane sections, which were mounted directly onto charged glass slides (Thermo Scientific) and allowed to dry for 2 h. TG sections were carefully rehydrated with 0.1 M PB (3 times) and incubated with 1% BSA and 1% Triton X-100 in 0.1 M PB for 1 h, and immunostaining was performed as described above. Tissue sections were mounted in FluorSave, a coverslip was applied, and images were acquired and analyzed. Slides were imaged using an automated AxioVision Observer Z1 (Zeiss) microscope or using a Zeiss LSM 880 with Airyscan. Images were acquired for analysis, and images were analyzed using ZEN2012 or NIH Fiji software.

### RNA isolation and real-time qPCR.

RNA was isolated by TRIzol extraction (Thermo Fisher) according to the manufacturer’s instructions. RNA was treated with the DNA-free kit (Ambion), and cDNA was synthesized using the SuperScript III reverse transcriptase kit (Invitrogen) with oligo(dT) (Promega) for real-time quantitative PCR (RT-qPCR). SYBR Select master mix (Life Technologies) was used, and RT-qPCR was performed using a CFX96 Touch real-time PCR detection system.

Oligonucleotides were as follows: β-actin, Fw, AGT GTG ACG TTG ACA TCC GT, and Rv, TGC TAG GAG CCA GAG CAG TA; Isg15, Fw, TGA GCA TCC TGG TGA GGA ACG AAA, and Rv, AGC CAG AAC TGG TCT TCG TGA CTT; Vps4A, Fw, GAC AAC GTC AAC CCT CCA GA, and Rv, AGC ATG CTG GTA GAG ACG GA; Vps4B, Fw, GCC TTG TCT GTA GTA GGG GAC, and Rv, TTC CCA GCT TTG TCT TCC TGG; Chmp4B, Fw, GCC CGA AAC AGT CCC TCT AC, and Rv, TTC CTT CTT CTT GGC GGG TT; Chmp2B, Fw, AAG CAG CTT GTC CAC CTA CG, and Rv, TTG CAT TGT CTT TGC AGT GGT.

### Statistical analysis.

Statistical analysis was performed using GraphPad Prism. For two group conditions, data were analyzed using unpaired *t* test. For multiple group conditions, two-way ANOVA followed by a Bonferroni posttest was performed.
